# Percutaneous Vertebroplasty With Cement-Augmented Hollow Screw Fixation for Kümmell Disease: A Case Report Highlighting Diagnostic Challenges and Surgical Outcomes

**DOI:** 10.7759/cureus.85731

**Published:** 2025-06-10

**Authors:** Anuj Kumar Pandey, Sachin, Faisal Kamal, Tuerhong Tuerxun

**Affiliations:** 1 Orthopedics, Xinjiang Medical University, Urumqi, CHN; 2 Spine Surgery, The 6th Affiliated Hospital of Xinjiang Medical University, Urumqi, CHN

**Keywords:** avascular osteonecrosis, back pain, percutaneous vertebroplasty, spine injury, vertebral body fractures

## Abstract

This case report presents a 79-year-old male with Kümmell disease (KD) who experienced progressive lower back pain following a minor fall. Imaging confirmed L1 vertebral collapse with osteonecrosis and posterior ligamentous complex (PLC) injury (thoracolumbar injury classification and severity score (TLICS) score=5). The patient underwent successful percutaneous vertebroplasty with cement-augmented hollow screw fixation under local anesthesia, resulting in immediate pain relief and restored spinal stability. This case highlights the importance of early surgical intervention in KD patients with spinal instability. Accurate diagnosis requires a detailed medical history and awareness of this rare condition. Treatment should involve careful observation of conservative methods' efficacy, and surgical planning should be considered after vertebral fracture deterioration to avoid adverse effects.

## Introduction

Kümmell disease (KD) is a rare condition characterized by vertebral collapse, typically in the thoracolumbar vertebrae, due to avascular necrosis of the vertebral body [1,2]. This often occurs weeks or months after a seemingly minor trauma in elderly patients [2]. Osteoporosis is the most significant risk factor, increasing the prevalence of KD in this population [3]. The patient was not formally diagnosed with osteoporosis. While osteoporosis is a significant risk factor for KD, its absence in this particular case highlights that KD can occur independently. This case report describes the diagnostic challenges and surgical management of KD in an elderly patient using percutaneous vertebroplasty combined with cement-augmented hollow screw fixation under local anesthesia [4]. This combined approach addresses both the osteonecrosis and the structural instability associated with this condition [4].

## Case presentation

Patient information and chief complaint

A 79-year-old married male presented with a three-month history of lower back pain that had worsened over the preceding three days. The patient's injury occurred from a slip and fall in the bathroom, landing on their lumbosacral region. Initially, he experienced severe pain and restricted movement, preventing ambulation. He denied having either loss of consciousness, numbness, or weakness in his limbs. Initial X-rays at a local hospital revealed a compression fracture of the L1 vertebra, and surgery was recommended but declined by the patient, who opted for conservative management. This included a thoracolumbar orthosis brace for spinal stabilization, oral analgesics for pain control, calcium and vitamin D supplementation, and activity modification with restricted mobility. An MRI confirmed damage to the anterior, middle, and posterior columns of L1, along with significant injury to the posterior ligamentous complex (PLC).

Three months later, his pain persisted and worsened, prompting him to seek further treatment at our institution. Upon admission, he complained of persistent lower back pain with restricted activity. Physical examination revealed tenderness to palpation over the L1 spinous process and paraspinal muscle spasm. Neurological examination was unremarkable.

Diagnosis 

**Figure 1:**
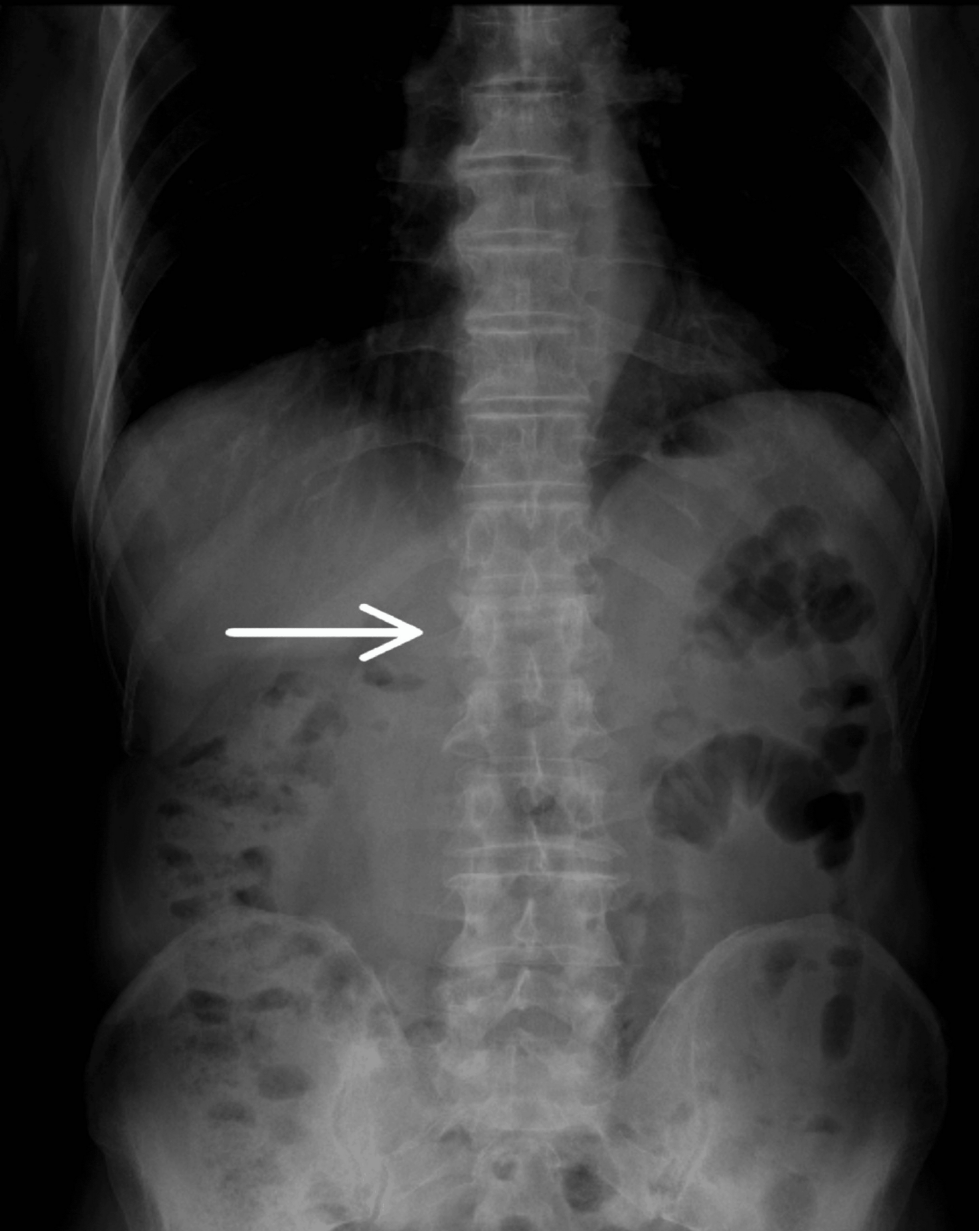
Anteroposterior X-ray view showing L1 vertebral fracture.

**Figure 2:**
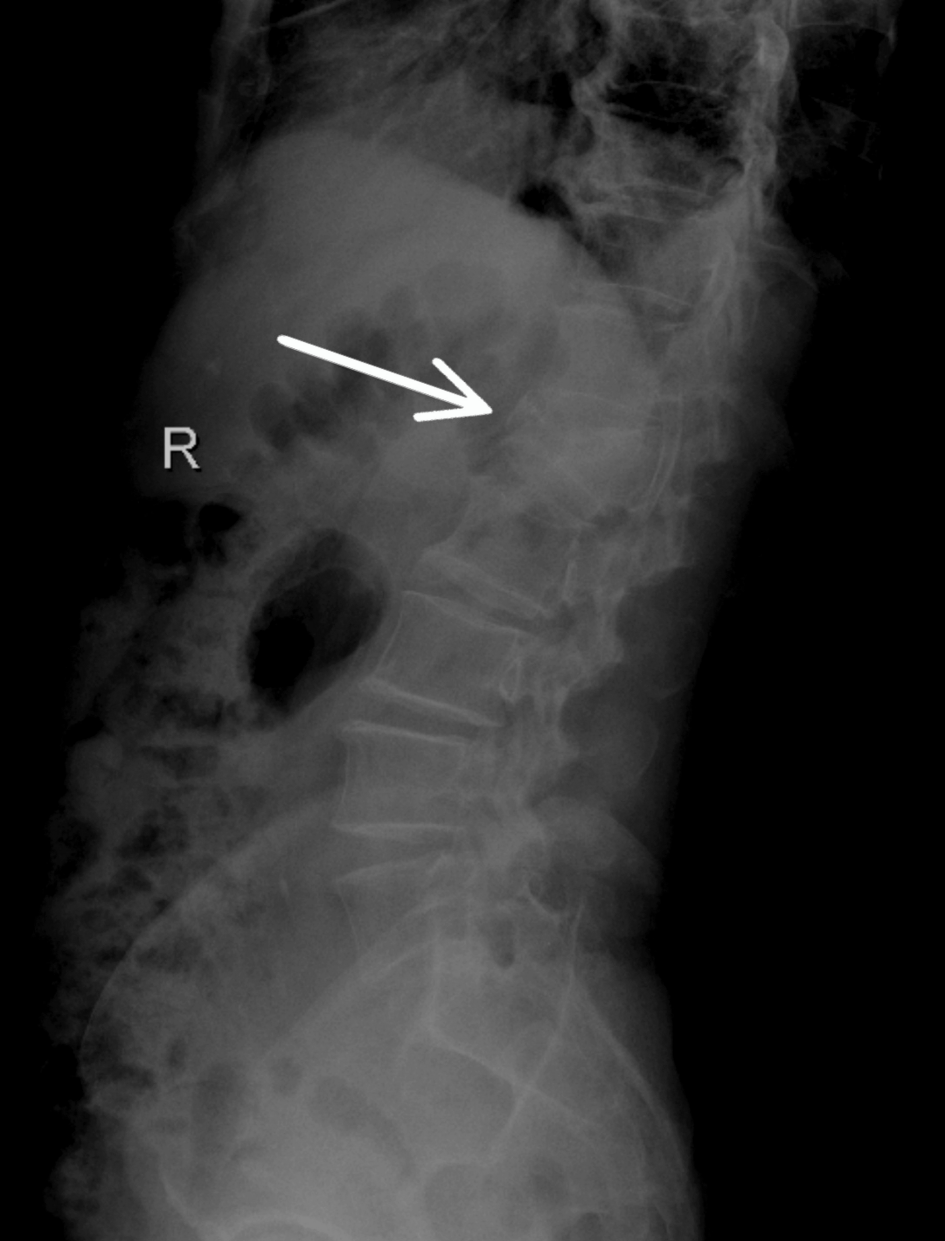
Lateral X-ray view showing L1 vertebral fracture.

**Figure 3:**
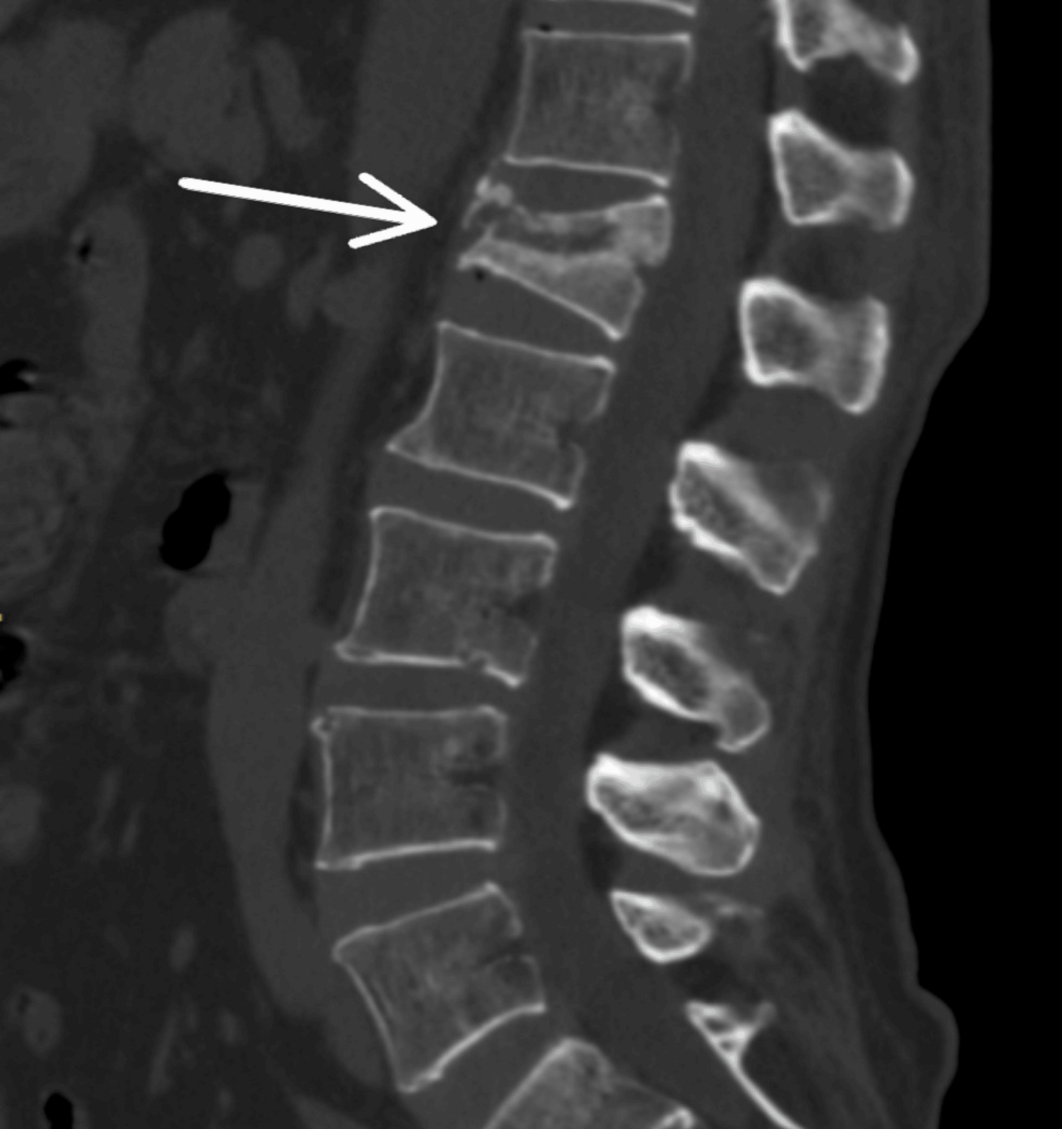
Lateral CT scan view showing L1 vertebral fracture.

**Figure 4:**
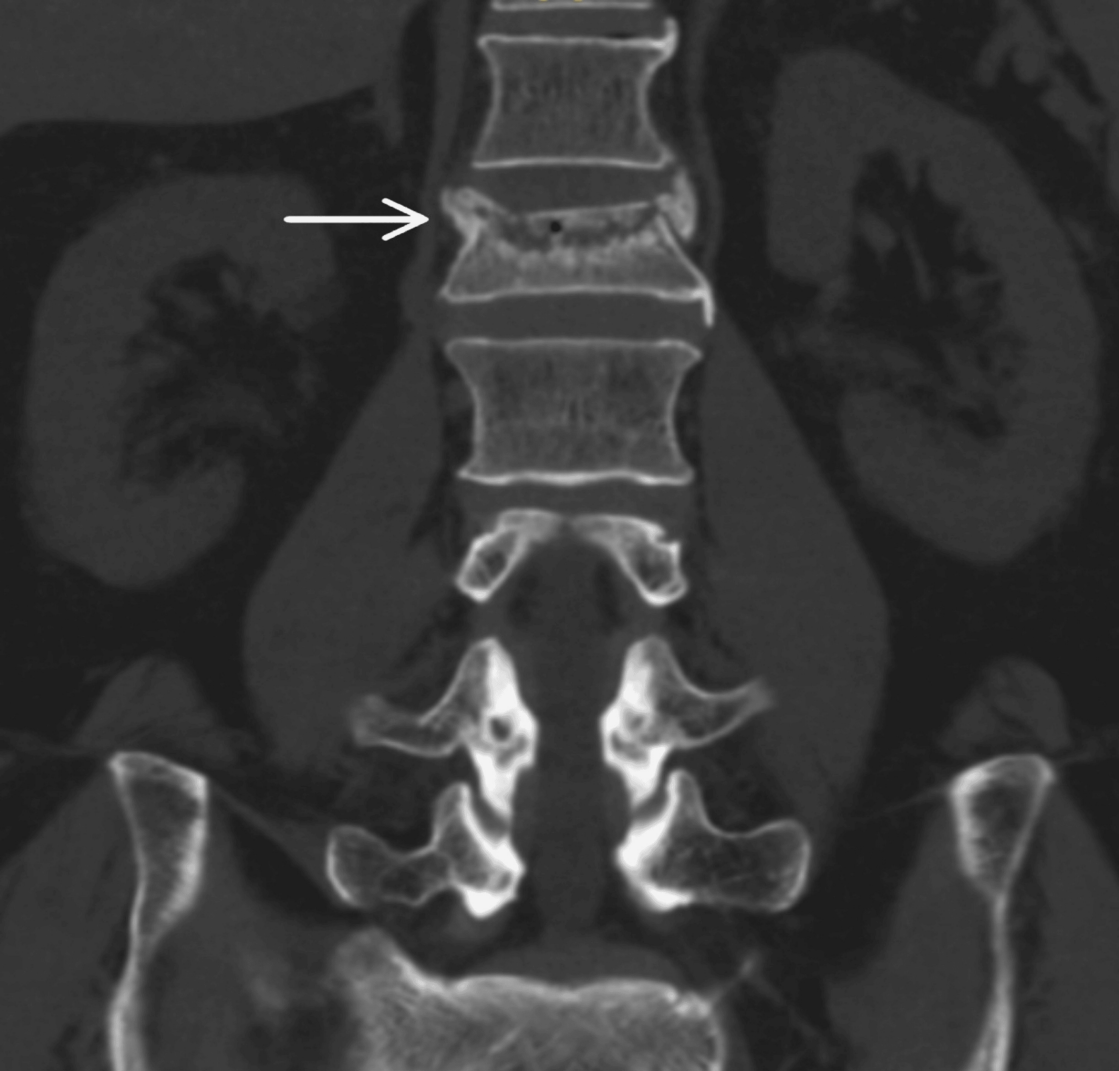
Anteroposterior CT scan view showing L1 vertebral fracture.

**Figure 5:**
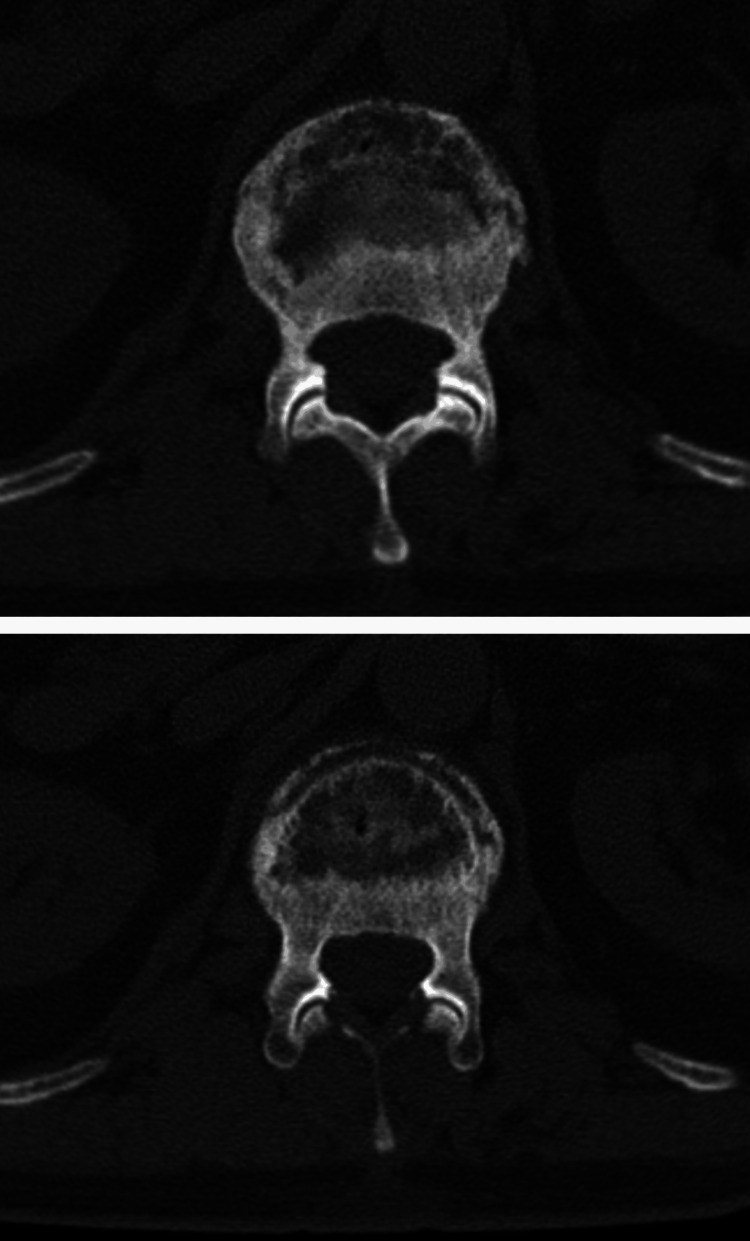
Axial (transverse) CT scan view showing L1 vertebral fracture.

Repeat X-rays (Figure 1 and Figure 2) confirmed the L1 compression fracture. A CT scan (Figure 3, Figure 4, and Figure 5) further delineated the fracture and revealed the presence of a vacuum cleft sign (Figure 5), providing further evidence of L1 vertebral bone osteonecrosis, solidifying the diagnosis of KD. Based on the Thoracolumbar Injury Classification and Severity Score (TLICS), the patient received 2 points for the burst fracture morphology and 3 points for PLC injury, resulting in a total score of 5, indicating spinal instability. Given his age, persistent pain, and progressive osteonecrosis, surgical intervention was recommended.

Interventions, follow-up, and outcomes

The patient was positioned prone on the operating table, and a C-arm was used to localize and mark the L5 vertebral body. After routine disinfection and draping, 1% lidocaine was injected into the lateral aspect of the L5 pedicle for local infiltration anesthesia. A fine needle was introduced for puncture, and localization was confirmed again with fluoroscopy to ensure the puncture needle was within the L5 vertebral body, followed by the injection of 5.0 mL of bone cement to diffuse within the vertebral body. One hollow screw, measuring 35-45 mm, was implanted through the pedicle for fixation. The patient reported slight, tolerable distension pain in the lower back. Postoperatively, the patient experienced no palpitations, chest tightness, or breathing difficulties and safely returned to the ward.

Following a percutaneous vertebroplasty, the patient exhibited a stable and progressive recovery. The surgery was uncomplicated, and the patient was transferred to the ward with stable vital signs (temperature: 36.3°C, pulse: 74 bpm, respiratory rate: 19 breaths per minute, blood pressure: 100/56 mmHg) and improved limb function compared to preoperative status. Postoperative care involved cardiac monitoring, oxygen, a gradual diet, and close observation of limb activity. Treatments focused on nerve health, gastric protection, circulation, and symptom relief.

**Figure 6:**
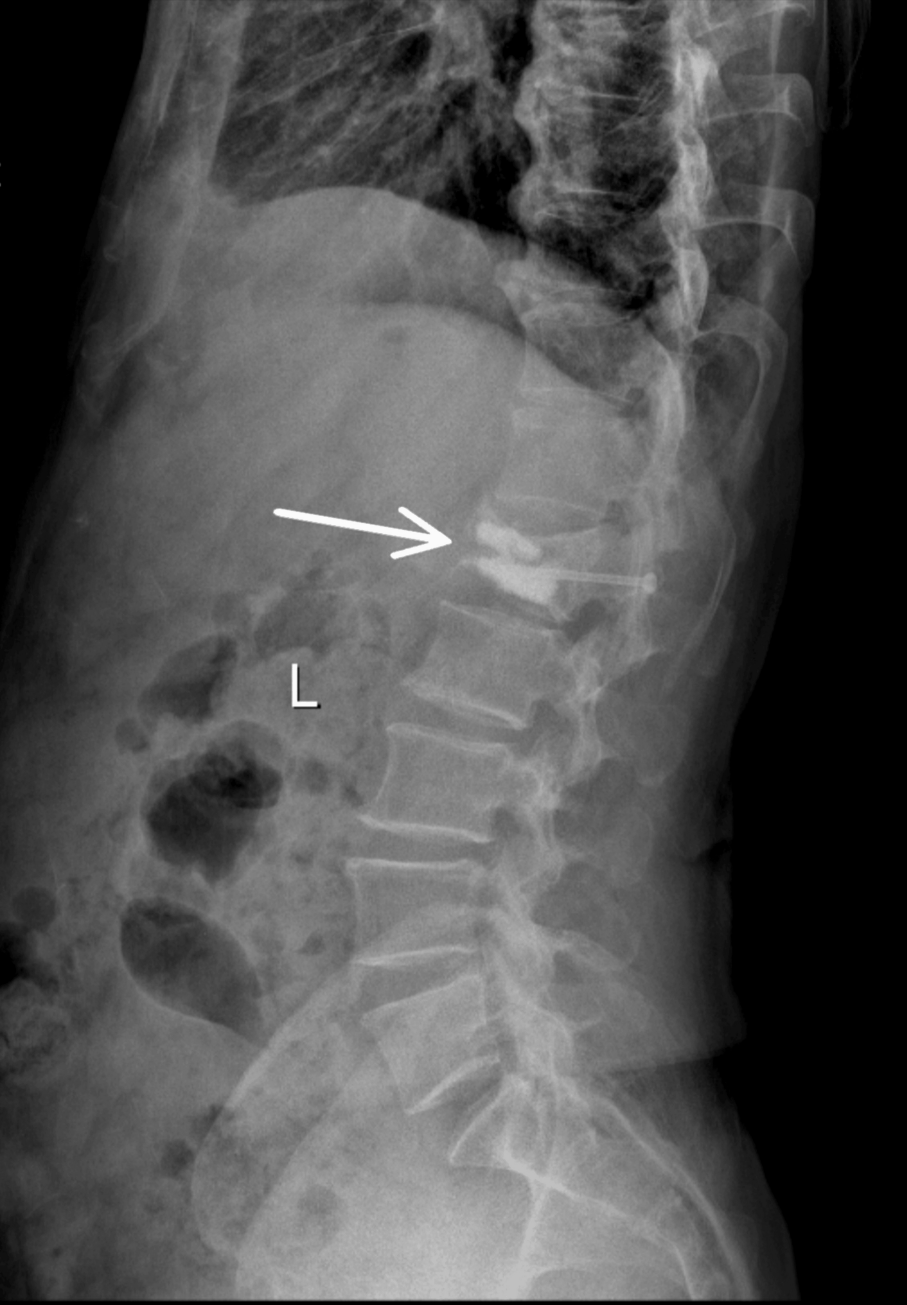
Lateral X-ray view of L1 vertebra after the surgical procedure.

**Figure 7:**
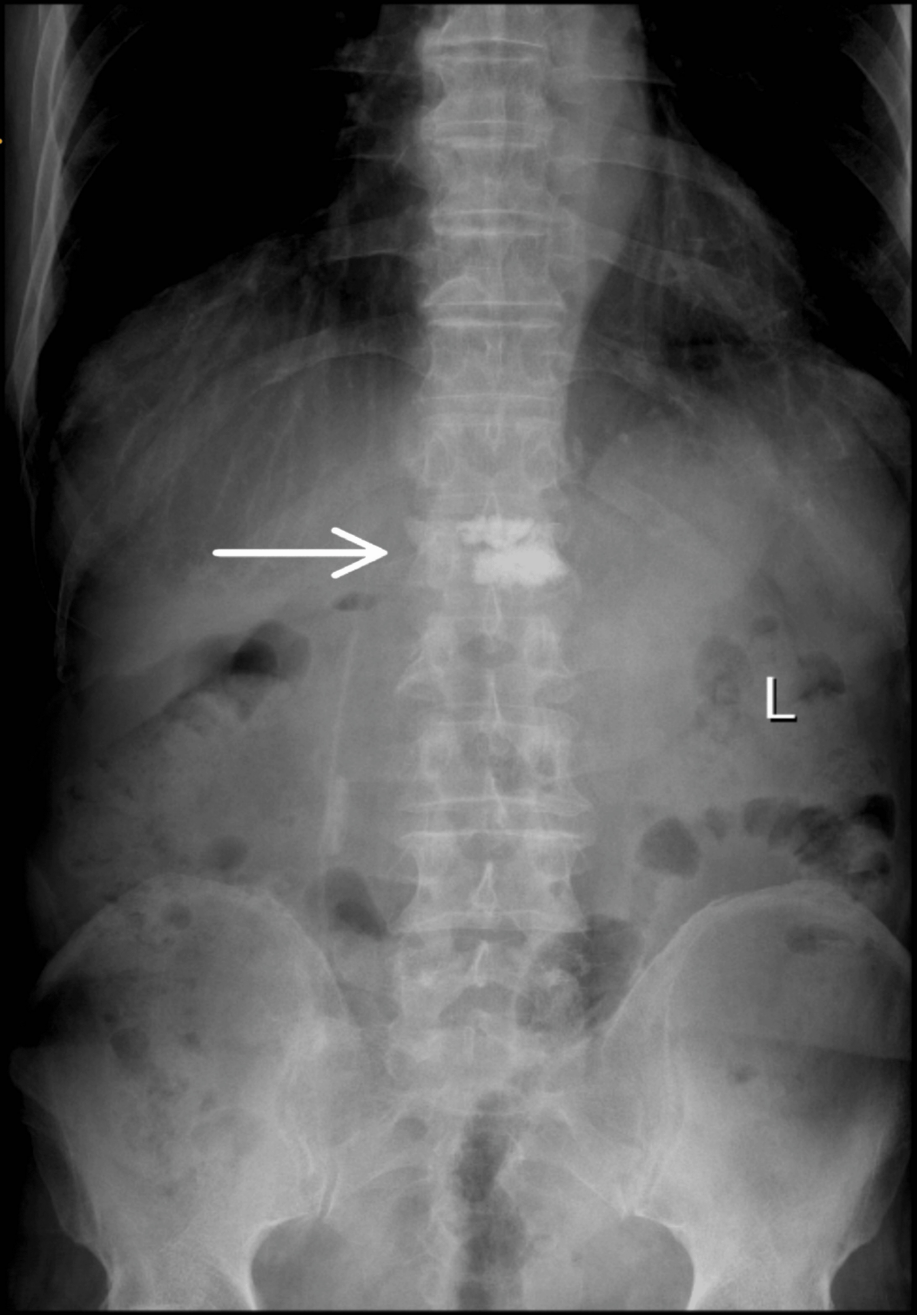
Anteroposterior X-ray view of the L1 vertebra after the surgical procedure.

The day after surgery, the patient reported reduced back pain and improved mobility. Mental status and basic functions were normal. Preventative measures for deep vein thrombosis were initiated, alongside traditional Chinese medicine therapies like hot packs and acupoint treatment, mud therapy, and ultrasound. Medications included intravenous fluids, dexamethasone, and oral blood tonic. The VTE risk was initially low, requiring monitoring. Two days post-surgery, the patient showed further improvement in pain and mobility, with a clean incision. Despite a medium VTE risk, the patient was cleared for discharge a few days later with instructions for three months of rest, avoidance of heavy lifting and cold exposure, engagement in supported moderate activity, and adherence to follow-up appointments. During the procedure, the hollow screw (Figure 6) and the radio-opaque cement within the body of the L1 vertebra (Figure 7) can be seen.

## Discussion

KD is an eponym for delayed post-traumatic bone osteonecrosis. Patients usually present with advanced-stage kyphosis in the thoracolumbar area within months to years after experiencing a minor trauma, initially presenting without any symptoms [2]. This case report highlights the successful application of percutaneous vertebroplasty combined with cement-augmented hollow screw fixation under local anesthesia in a 79-year-old male with KD-induced L1 vertebral collapse and significant PLC injury (TLICS score of 5), resulting in spinal instability. This combined approach directly addressed both the avascular necrosis and the structural compromise, leading to immediate pain relief and restored stability. The diagnostic pathway for KD necessitates a high index of suspicion, particularly in elderly patients presenting with persistent or worsening back pain following a seemingly insignificant fall [5]. One of the key diagnostic challenges lies in differentiating KD from typical osteoporotic vertebral compression fractures (OVCFs), especially in the early stages. While initial X-rays may reveal a compression fracture, as seen in this case, the delayed onset of vertebral collapse and osteonecrosis characteristic of the progressive nature of KD often requires further investigation with advanced imaging, such as CT and MRI [6]. The CT scan in our patient was crucial in delineating the fracture morphology and revealing the osteonecrosis of the L1 vertebral body, confirming the diagnosis of KD. Importantly, CT scans can also reveal the vacuum cleft sign, a key indicator of non-union and fluid accumulation within the fractured vertebra, strongly suggestive of KD [7]. Furthermore, the MRI findings of significant PLC injury underscored the spinal instability, necessitating surgical intervention. Currently, the literature lacks standardized treatment protocols for KD [8], underscoring the need for more robust evidence, particularly from randomized controlled trials, to guide clinical decision-making. While minimally invasive surgery (MIS) has demonstrated efficacy in treating OVCFs [9], the compromised healing potential in KD [10] warrants careful consideration when contemplating invasive procedures. Although surgical stabilization is considered for OVCFs with nonunion and persistent pain [11], conservative treatment for KD often has limited efficacy as vertebral body necrosis tends to progress, and natural healing is unlikely. In such cases, surgical intervention is frequently recommended, especially when neurological deficits are present [12]. In this specific case, the patient's persistent and worsening pain, coupled with the evidence of progressive osteonecrosis and spinal instability (TLICS: 5), prompted the decision for surgical intervention after the initial conservative recommendation was declined. The chosen technique, percutaneous vertebroplasty with cement-augmented hollow screw fixation, offered a minimally invasive solution to stabilize the fractured vertebra and address the underlying osteonecrosis by providing structural support with the cement. The use of hollow screws allowed for cement augmentation, enhancing the fixation strength in the osteoporotic bone, a crucial factor in elderly patients [13]. Furthermore, performing the procedure under local anesthesia minimized the risks associated with general anesthesia in this elderly patient. This case report highlights a potentially novel approach to treating KD with significant PLC injury and instability (TLICS 5) in a high-risk elderly patient [14]. The combination of vertebroplasty and cement-augmented hollow screw fixation, performed as a single-stage, minimally invasive procedure under local anesthesia, appears to address both the osteonecrosis characteristic of KD and the instability caused by the PLC damage.

This approach may differ from existing literature in several ways. Prior studies often describe vertebroplasty as a standalone treatment for KD, particularly in cases without significant instability, focusing on pain relief and vertebral height restoration through vertebroplasty or kyphoplasty. However, in cases with substantial instability due to posterior ligamentous complex (PLC) injury, traditional approaches usually favor open surgical stabilization, often requiring general anesthesia, with literature commonly discussing open reduction and internal fixation techniques for vertebral compression fractures (VCFs) with PLC injury. In contrast, the described case employs a minimally invasive approach to manage a complex scenario involving both osteonecrosis and significant instability. While some studies support minimally invasive methods like percutaneous vertebroplasty for KD, others recommend open surgery for unstable fractures [15]; the combination of minimally invasive stabilization with cement augmentation in this context is less frequently reported. Additionally, performing this combined procedure under local anesthesia in an elderly, high-risk patient represents a notable deviation from standard practices, as stabilization procedures are typically conducted under general anesthesia.

The successful outcome reported in this case suggests that this combined, minimally invasive technique under local anesthesia could be a valuable option for carefully selected elderly patients with KD and instability, offering the benefits of immediate pain relief, enhanced stability, and reduced surgical morbidity compared to more invasive open procedures. Further research and comparative studies would be needed to validate these findings and define the specific indications for this nuanced approach.

The positive outcomes observed in our patient, including immediate pain reduction and enhanced mobility following surgery, are consistent with existing literature supporting the use of vertebroplasty in the management of VCFs [16]. Cement augmentation, which was utilized to stabilize the PLC injury in our case, directly addressed the source of spinal instability. Surgical intervention in patients with KD and demonstrated spinal instability can effectively alleviate pain and improve spinal stability, potentially halting progressive vertebral deterioration and averting associated complications [17]. While some studies debate the long-term efficacy of vertebroplasty alone [18], this case highlights the potential benefit of a combined approach in the presence of significant instability.

This case underscores the importance of considering surgical options for KD patients who display worsening vertebral integrity and spinal instability, even in the absence of neurological deficits [17]. While initial conservative management may be appropriate, vigilant monitoring and a readiness to pursue surgical intervention when necessary are critical for optimizing patient outcomes and preventing adverse sequelae [19]. Further investigation is needed to establish definitive treatment algorithms for KD and to comprehensively assess the long-term efficacy of various surgical techniques, particularly the combined approach employed in this instance, through larger, controlled studies [20].

## Conclusions

In this case, the patient successfully recovered after the surgical procedure of hollow screw and cement placement in the L1 vertebra, with no further pain. Accurate diagnosis of KD requires a detailed medical history and awareness of this rare condition. Treatment should primarily involve careful observation of the efficacy of conservative methods, and surgical planning should be considered after any deterioration of vertebral fractures to avoid adverse effects. Further research is needed to fully demonstrate the effectiveness of various surgical techniques in treating KD, as well as to assess the risk of complications and the long-term outcomes of these procedures.
